# The Impact of Heatwaves on Community Morbidity and Healthcare Usage: A Retrospective Observational Study Using Real-Time Syndromic Surveillance

**DOI:** 10.3390/ijerph13010132

**Published:** 2016-01-16

**Authors:** Sue Smith, Alex J. Elliot, Shakoor Hajat, Angie Bone, Chris Bates, Gillian E. Smith, Sari Kovats

**Affiliations:** 1 Real-Time Syndromic Surveillance Team, Public Health England, Birmingham B3 2PW, UK; sue.smith@phe.gov.uk (S.S.); gillian.smith@phe.gov.uk (G.E.S.); 2 NIHR Health Protection Research Unit in Environmental Change and Health, London School of Hygiene and Tropical Medicine, London WC1H 9SH, UK; shakoor.hajat@lshtm.ac.uk (S.H.); sari.kovats@lshtm.ac.uk (S.K.); 3 Extreme Events and Health Protection, Public Health England, London SE1 8UG, UK; angie.bone@phe.gov.uk; 4 ResearchOne, The Phoenix Partnership, Leeds LS18 5TN, UK; chris.bates@tpp-uk.com

**Keywords:** heatwave, general practitioner, emergency department, telehealth, syndromic surveillance

## Abstract

We investigated the impact of a moderate heatwave on a range of presenting morbidities in England. Asthma, difficulty breathing, cerebrovascular accident, and cardiovascular symptoms were analysed using general practitioner in hours (GPIH), out of hours (GPOOH) and emergency department (ED) syndromic surveillance systems. Data were stratified by age group and compared between a heatwave year (2013) and non-heatwave years (2012, 2014). Incidence rate ratios were calculated to estimate the differential impact of heatwave compared to non-heatwave summers: there were no apparent differences for the morbidities tested between the 2013 heatwave and non-heatwave years. A subset of GPIH data were used to study individuals at higher risk from heatwaves based on their pre-existing disease. Higher risk patients were not more likely to present at GPs or ED than other individuals. Comparing GPIH consultations and ED attendances for myocardial infarction/ischaemia (MI), there was evidence of a fall in the presentation of MI during the heatwave, which was particularly noted in the 65–74 years age group (and over 75 years in ED attendances). These results indicate the difficulty in identifying individuals at risk from non-fatal health effects of heatwaves and hot weather.

## 1. Introduction

Heatwaves impact on community morbidity and mortality in the United Kingdom (UK) [1,2,3]. Exposure to high temperatures can cause a range of heat conditions such as heat exhaustion or heatstroke, but also cardiovascular disease, renal disease and respiratory problems, in turn leading to excess emergency department attendances and ambulance calls-outs [4]. In the UK, general practitioner (GP) consultations for heat conditions have been shown to increase during heatwaves [5]. Heatwaves and hot weather are also associated with increases in daily mortality (heat-related mortality) [6,7,8,9,10]. During the major European heatwave of August 2003, excess mortality was recorded across western and central Europe [11]. In England, there were 2091 excess deaths during the 2003 heatwave and excess hospital admissions of 16 per cent [12,13]. France reported over 15,000 excess deaths during a 16 day period of high day and night temperatures, the majority occurring in the elderly [14,15].

Climate change is very likely to increase the likelihood of hotter summer and severe heatwaves [16,17]. Projections of temperature related mortality show significantly raised risk of heat-related deaths across all regions of England in the coming decades which will have implications for public health and lead to acute pressures on health services [18]. Current measures to protect the general public from the health effects of extreme hot weather are based on the Heatwave Plan for England and include key health advice to the public about keeping cool, advice to healthcare providers about protecting patients and engaging with community groups to protect vulnerable sections of the population [19]. The Plan was established in 2004 in response to the 2003 European heatwave.

Syndromic surveillance is the near real-time collection, analysis, interpretation and dissemination of health-related data to enable the early identification of the impact (or absence of impact) of potential public health threats which require effective public health action [20]. Previous work has illustrated the usefulness of using syndromic surveillance for monitoring patients presenting with heatstroke during periods of heat [5,21,22,23,24,25]. However, one limitation of this approach is that the numbers of heatstroke cases are proportionally low, especially when analysing data further by age or geographic region. Therefore, the aim of this work was to consider the potential sensitivity of more common morbidity outcomes to inform the planning and response to heatwaves in England. In addition, it has also been difficult to identify high risk individuals for interventions for heat health protection. Therefore a second aim was to examine a subset of high risk individuals to see if they were more likely to indicate an increase in heat-related morbidity. We used syndromic surveillance to achieve these aims as these systems are able to provide real-time intelligence during a heatwave, a comprehensive picture of national activity, historic data can be used to identify current anomalies and they can be used to monitor a range of morbidity indicators in the general population.

## 2. Experimental Section

### 2.1. Study Period

Data were included for the period 1 June to 15 September for the years 2012–2014. This period was chosen as it coincided with the operation of the summer Heat-Health Watch alert system in England [26]. The Heat-Health Watch alert system operates each summer and comprises five main levels: long-term planning (level 0); heatwave and summer preparedness (level 1); alert and readiness (level 2); heatwave action (level 3); national emergency (level 4). Each alert level is underpinned by a series of trigger temperatures (day-time and night-time), which are based upon temperatures where there is increased risk of impacts on health [19]. A heatwave occurred during July 2013 in England resulting in a series of level 3 alerts (indicating a heatwave and initiating a public health response). Unusually hot weather affected most of the UK from 3 to 23 July 2013, making the month the 3rd hottest July on record [27]. Within that period there were 13 days, from 11–23 July, when the heat health alert level remained at level 2 or above. The years 2012 and 2014 were selected as comparison years as there had been no significant heatwave activity and they were included in the historical data of the syndromic surveillance systems used in the study.

### 2.2. Syndromic Surveillance Systems

Public Health England (PHE) coordinates a number of national syndromic surveillance systems and delivers a real-time syndromic surveillance service that has been described in detail elsewhere [28]. In brief, anonymised data for a range of different signs and symptoms are collected on a daily basis from a number of healthcare service providers across England. These signs and symptoms are aggregated into a number of syndromic indicators, e.g., gastroenteritis based upon symptoms and clinical diagnoses of disease. Data capture, analysis and statistical interpretation of daily syndromic surveillance data have been described in detail elsewhere [28,29]. In summary, anonymised data are received from a number of health care providers using secure automated transfer processes on a daily basis. Data are interrogated using a range of epidemiological and statistical algorithms to determine recent trends and statistical exceedances.

Morbidity data from a suite of national syndromic surveillance systems were used, including general practitioner in hours (GPIH), GP out of hours (GPOOH) and emergency department (EDSSS) syndromic surveillance systems (Table 1). Emergency department (ED) attendance data from 18 EDs that had reported to the EDSSS continuously for the years 2012–2014 were compared.

**Table 1:** Syndromic surveillance systems used in the study and the syndromic indicators included within the analysis.

Surveillance System	Approximate Population Coverage (% of England Population)	Syndromic Indicator		
		Cardiovascular	Respiratory	Others
**GP in hours (GPIH)**	35 million (64%)	Myocardial infarction *	Asthma, wheeze	Cerebrovascular accident *
**GP out of hours (GPOOH)**	33 million (60%)	N/A	Asthma, Asthma/Wheeze/Difficulty Breathing	Fever, rash, cerebrovascular accident
**Emergency department (EDSSS)**	35 sentinel EDs **^†^**	Myocardial ischaemia	Asthma, Asthma/Wheeze/Difficulty Breathing	Cerebrovascular accident

* these indicators are not routinely used in the daily syndromic surveillance and are derived from a subset of the GPIH system with a denominator of approximately 5 million patients. **^†^** Population coverage not possible due to difficulties in calculating denominator population. N/A no indicators available.

### 2.3. Syndromic Surveillance Data Analysis

It is known that mortality from respiratory and cardiovascular causes increases at high temperatures [1,30,31]. Therefore, we used a corresponding suite of syndromic surveillance morbidity indicators (based upon an aggregation of underlying clinical codes) based upon these potential heat-related clinical links (Table 1). 

GPIH consultations were presented as the daily rate per 100,000 patient population using numbers of consultations (numerator) and registered patient populations (denominator). It was not possible to calculate accurate patient denominators for the GPOOH and EDSSS systems due to problems defining the “catchment population” for individual hospitals/EDs, and individual GPOOH service providers. Therefore, for the EDSSS and GPOOH systems a daily percentage of all recorded contacts (attendances/consultations) was calculated for each syndromic indicator.

### 2.4. Meteorological Data Sources

Central England Temperature (CET) data (mean daily °C) were obtained from the UK Met Office Hadley Centre observation dataset [32]. The heatwave period was defined as 3–23 July 2013 based upon alert criteria published in the Heatwave Plan for England, and the alerts given during the 2013 heatwave [5,19]. 

### 2.5. Trend Analyses

Time series graphs illustrating daily data for syndromic indicators (including seven day moving averages of rates/percentages) were plotted for the GPIH, GPOOH and EDSSS surveillance systems. Trends were initially examined visually to identify whether indicators increased during summer/heatwave periods, and further compared to other years to identify inter-seasonal differences. Trends were also compared with daily CET data. Syndromic surveillance data were further analysed stratified by age (age groups 0–4, 5–14, 15–64, 65–74, and ≥75 years). 

### 2.6. Incidence Rate Ratios

Incidence rate ratios (IRRs) were calculated for the GPIH system using the mean weekly incidence rate (per 100,000 population) for syndromic indicators for weeks 28–30 (8 July through 28 July 2013) when the alert reached level 2 or 3 during the heatwave period of 2013. The calculation of weekly IRRs was repeated for the same period in the non-heatwave years 2012 and 2014. An IRR was calculated by comparing the mean values for the heatwave and non-heatwave years and was stratified by age (age groups 0–4, 5–14, 15–64, 65–74, and ≥75 years). Further stratification by sex was not undertaken due to resulting small numbers. The Taylor method was used for calculating 95% confidence intervals (CIs) for the IRR [33], with the assumption that there was independence between years so that the covariance term was zero. A similar method was applied to the EDSSS and GPOOH systems using the percentage of attendances/consultations to develop a percentage ratio. 

### 2.7. GPIH Risk Group Analysis

A subset of the GPIH data, based upon a denominator of approximately 5.5 million patients, were analysed by risk group to determine whether patients in those groups were more susceptible in hot weather. In the UK, annual seasonal influenza vaccination is recommended to patients with a defined health risk, including asthma, coronary heart disease, diabetes, immunosuppression, kidney disease, liver disease, neurological and respiratory disease [34,35]. For the purposes of this study we therefore included influenza vaccination indication (however not including pregnancy, age or carer status) as a proxy for patients with underlying comorbidities who were at risk from the impact of heat. At risk patients were defined as those presenting with a diagnosis included within a syndromic indicator, with any of the influenza vaccine clinical risk groups flagged on their electronic GP patient record (based upon clinical Read codes [36]). 

The measure of risk for each morbidity indicator was calculated as the percentage of risk patient consultations compared to the total patient consultations. The percentage risk group consultations was calculated over the heat health watch period (1 June to 15 September) for each of the years 2012–2014 for selected indicators.

## 3. Results and Discussion

### 3.1. General Practitioner in Hours Consultations

**Figure 1:**
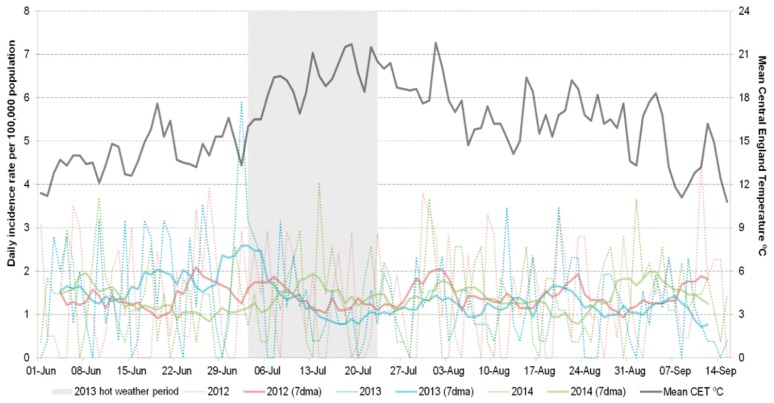
Daily GPIH consultations for myocardial infarction (65–74 years).

Overall, none of the indicators showed a better response to the heatwave than the heatstroke indicator currently used in the heatwave surveillance system [5]. Respiratory indicators, including severe asthma and wheeze, showed no change in trend during the 2013 heatwave period. Rates of cerebrovascular accident (CVA) and myocardial infarction did not increase during the 2013 heatwave and were similar to other years, however GPIH myocardial infarction consultations in the 65–74 years age group decreased over the heatwave period as the temperature increased (Figure 1). The weekly incidence rate ratio (IRR) for weeks 28 to 30 (8 July through 28 July 2013) showed a 23% decrease compared to the same period for 2012 and 2014 combined, although this result was not significant (IRR 0.77 (CI 0.30–1.23); Table 2). 

**Table 2:** Incidence Rate Ratios (IRRs) comparing mean incidence of GPIH myocardial infarction (mean weekly rate) and EDSSS myocardial ischaemia (mean weekly % attendances) for weeks 28–30 of 2013 compared to the same period in 2012 and 2014.

Age Group	GPIH Myocardial Infarction Incidence Rate Ratio (95% CI)		EDSSS Myocardial Ischaemia Incidence Rate Ratio (95% CI)	
0 to 4	–	–	–	–
5 to 14	0.00	–	–	–
15 to 64	0.92	(0.70–1.14)	0.88	(0.50–1.27)
65 to 74	0.77	(0.30–1.23)	0.88	(0.46–1.29)
75 plus	1.07	(0.80–1.34)	0.75	(0.65–0.84)
all ages	0.92	(0.75–1.09)	0.84	(0.57–1.11)

### 3.2. General Practitioner in Hours Syndromic Indicators by Risk Group

For asthma, myocardial infarction and CVA indicators there was no significant difference (indicated by overlapping confidence intervals) in the percentage of consultations in a risk group during the 2013 heat health period compared to the same period for the years 2012 and 2014 (Table 3). The percentage of wheeze cases occurring amongst those patients in a risk group showed a significant increase each year from 2012 to 2014, however summer 2014 was cooler than 2013 so this increase was unlikely to be related to heat, and may have been due to other underlying consultation and coding practices. 

### 3.3. General Practitioner Out of Hours Consultations

**Figure 2:**
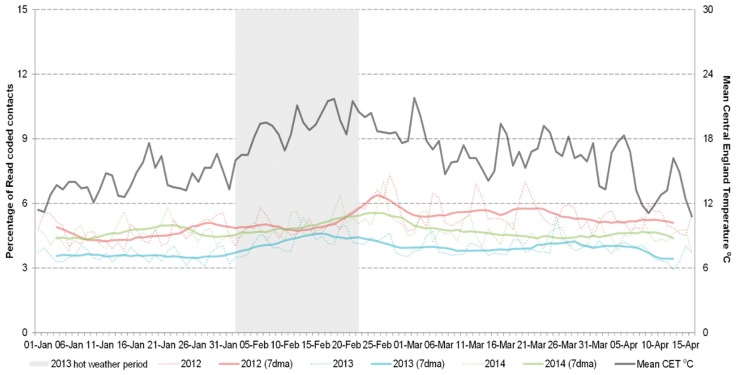
Daily GPOOH consultations for rash 15–64 years (including seven day moving average) 2012–2014.

GPOOH consultations for difficulty breathing/wheeze/asthma showed no increase during the 2013 heatwave period, with trends being comparable with the other years. Similarly there were no unusual trends for asthma, trauma, fever or CVA consultations. Although fever increased in children during the heatwave, particularly the 5–14 age group, this was at the same time as the seasonal rise seen in other years and consistent with previous findings (data not presented) [24]. There was an increase in consultations for rash mainly in adults aged 15–64 years that coincided with the heatwave period, however the overall level of rash consultations during 2013 was lower than other years suggesting a change in coding practice by OOH services during that period (Figure 2).

**Table 3:** GPIH syndromic indicators by risk group (as % of all cases) for the period 1 June to 15 September in the years 2012–2014.

Indicator	2012			2013			2014		
	Risk Group	No Risk	% Cases in Risk Group (95% CI)	Risk Group	No Risk	% Cases in Risk Group (95% CI)	Risk Group	No Risk	% Cases in Risk Group (95% CI)
Asthma	1939	1358	59 (57.1–60.5)	1948	1028	65 (63.7–67.2)	2296	1089	68 (66.2–69.4)
Myocardial infarction	515	1128	31 (29.2–33.6)	692	1229	36 (33.9–38.2)	675	1232	35 (33.3–37.6)
Cerebrovascular accident	708	885	44 (42.0–46.9)	840	1053	44 (42.2–46.6)	868	1011	46 (44.0–48.5)
Wheeze	3290	7025	32 (31.0–32.8)	3564	6300	36 (35.2–37.1)	3887	6137	39 (37.8–39.7)

### 3.4. Emergency Department Attendances

**Figure 3:**
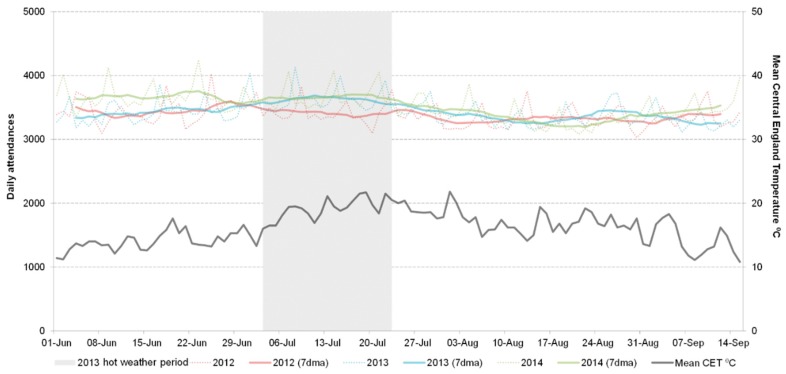
Total daily emergency department attendances reported to the EDSSS (including seven day moving average) 2012–2014 including 18 emergency departments reporting to the EDSSS 2012.

Emergency department attendances for all causes were similar in the 2013 heatwave to those in 2014, however, both of these years had higher attendances compared to 2012 (Figure 3).

There were no unusual trends in attendances for asthma, asthma/wheeze/difficulty breathing or CVA during the 2013 heatwave compared to 2012 and 2014. 

Attendances for myocardial ischaemia were lower during the 2013 heatwave period than 2012 and 2014. The weekly IRR for weeks 28 to 30 (8 July through 28 July 2013) for the 75 years and over age group showed a significant decrease of 25% compared to the same period for 2012 and 2014 combined (IRR 0.75 (CI 0.65–0.84); Table 2).

## 4. Conclusions

### 4.1. Main Findings

This paper explores the utility of real-time syndromic surveillance systems for monitoring the public health impact of hot weather using a range of health outcomes. The results of this current study illustrate that syndromic surveillance systems monitoring signs and symptoms of respiratory complaints such as asthma, wheeze and difficulty breathing were not sensitive to periods of hot weather. In addition, there was no evidence of increased incidence of CVA or myocardial infarction/ischaemia during the 2013 heatwave period compared to other years. This is consistent with other studies using hospital admissions data which has shown the MI risk increase with cold but not with heat [37]. Despite a small increase in GPIH myocardial infarction consultations prior to the heatwave period, as temperatures increased there was an overall decrease in GPIH consultations and emergency department attendances for myocardial infarction/ischaemia during the heatwave period, which was particularly noted in the 65–74 years age group and the 75 years and over in the ED attendances. There is still some uncertainty surrounding the link between heat and myocardial infarction; stronger links with mortality have been described [38]. Additionally, the contemporaneous recording of death in GP records is not consistent, or timely, and the cause of death may be attributed to other causes. Patients are less likely to present to routine GPIH services with acute myocardial infarction and therefore the GPIH data may monitor more routine patient management of chronic symptoms, or less severe cases. Both scenarios therefore might result in the heatwave having an indirect protective effect on patients with underlying co-morbidities: the heatwave plan advises this patient group to “stay out of the heat”, keep cool and not participate in strenuous activities [19], which may encourage patients to reschedule appointments until after the heatwave alert thereby resulting in lower rates of presentation. This however does not account for the decrease in myocardial ischaemia ED attendances reported and this requires further work to elucidate the underlying drivers for this fall.

### 4.2. How does This Work Compare to Others?

Studies have demonstrated increases in morbidity during heatwaves, including CVA and respiratory ambulance attendances [39], CVA hospitalizations [40] , respiratory admissions [41,42] and cardiac arrest (but not MI) [43]. The lack of impact shown in this current study may be explained by the relative mild temperatures and short exposure to heat experienced in the UK heatwave compared to other countries, and therefore there is less potential to impact on health. This is supported by a retrospective analysis of mortality data from the 2013 heatwave, which revealed that during the heatwave there was no excess in all-cause mortality, supporting the idea that this heatwave had less impact [44]. The observation of a decrease in MI in both the GP and ED systems is also supported by work from Germany and Czech Republic where excess mortality from MI was not accompanied by increases in hospital admissions [45,46].

### 4.3. Limitations

There are some potential limitations to this work. The two “non-heatwave” years selected contained a small number of level 2 alerts, which may have limited the differences when comparing to the 2013 heatwave year. However, there were no level 3 alerts or sustained periods of heat in either non-heatwave year. We used the classification of risk status based upon the conditions included in the influenza vaccination risk groups. Although in general this includes the majority relevant risk categories it may be too broad and further sub-analysis by individual risk group e.g., diabetes, renal disease may have provided more sensitive results. However, using a subset of GPIH data for this analysis generated small numbers of consultations by individual risk group and this was considered unsuitable for analysis. We did not undertake an analysis of sex-stratified data for the risk analysis, again due to the generation of small numbers unsuitable for analysis. Finally, the England heatwave of 2013 was relatively moderate compared to other documented heatwaves, both in maximal temperatures reached, and the duration of the increased period of heat. The results presented therefore do not account for more severe heatwaves which might result in different morbidity profiles presenting to health care services. The syndromic surveillance systems currently used by PHE were not operational in 2003 and therefore historical data covering more severe heatwaves were not available for analysis. This highlights the importance of having long running systems with rich historical datasets to cover such eventualities.

### 4.4. Clinical Implications

Previous work has illustrated the increased presentation of patients with heatstroke symptoms during heatwaves. This current work illustrates a lack of significant burden on health care services through presentation of other morbidities during a moderate heatwave. We also analysed patients with underlying risk comorbidities to ascertain whether this group had a higher rate of presentation than non-risk groups: findings illustrated that there were no differences for those morbidities tested. It can be concluded therefore that syndromic surveillance of morbidities other than specific heat/sun stroke symptoms does not add additional intelligence to the estimation of heat impacts. This has some relevance to the Heatwave Plan for England, which focuses primarily on the protection of risk groups from the effects of heat, and health care services who also target at risk groups including the elderly. As discussed in the limitations, however, this finding must be interpreted with some caution. The small subset of risk data did not allow for analysis of individual risk groups, which may have limited the usefulness of this approach. A future analysis would require a larger national morbidity database, and possibly analyzing data from a larger number of years to increase the power of the analysis. In addition, the impact during the 2013 heatwave, which was relatively short with moderately high temperatures had less impact than a more severe heatwave. It is likely that a prolonged period of heat, with high day and night temperatures would increase the impact in at risk groups and therefore increase the pressure on health services.

The aim of this work was ascertain whether current burden calculated using heath care presentation of heatstroke was in fact an underestimate of the public health impact of heat due to presentation of other heat-related symptoms. A limitation of this work is that the relationships described here are associated, and there is no causal link between the periods of heat and the cases of morbidity. However, syndromic surveillance data represent a unique opportunity to assess changes in patients presenting to health care services, at the population level, with a range of morbidities. 

### 4.5. Implications for Heatwave Plans

The results from this work suggest that there is no additional benefit in monitoring a range of health morbidity outcomes in addition to heatstroke symptoms within the England heatwave plan surveillance system. Previous work has demonstrated that monitoring the presentation of heatstroke symptoms provides a good indicator of the impact of heat across different health systems in the community [5], and in conjunction with these findings this will continue to be the main outcome measure and input to the heatwave plan. Monitoring mortality rates during heatwaves still represents the most effective measure of the severe impact of heat and systems are in place within England to monitor all-cause mortality during heatwaves in near real-time [47].
